# Muscle activation time and free-throw effectiveness in basketball

**DOI:** 10.1038/s41598-021-87001-8

**Published:** 2021-04-05

**Authors:** Paweł Pakosz, Przemysław Domaszewski, Mariusz Konieczny, Dawid Bączkowicz

**Affiliations:** Faculty of Physical Education and Physiotherapy, Opole University of Technology, 45-758, Opole, Poland

**Keywords:** Musculoskeletal system, Learning and memory

## Abstract

This study attempts to analyze the relationship between free-throw efficiency and the time of arm muscle activation in players from 3 basketball teams with different levels of experience was investigated. During the experiment each player made 20 free throws during which the activation time of his right and left biceps and triceps brachii muscles were measured with the use of surface electromyography and high-speed cameras. Significant differences in muscle activation time (t) during a free throw were found between the groups of basketball players (p = 0.038) (novices: t = 0.664 ± 0.225 s, intermediate-level players: t = 1.15 ± 0.146 s, experts: t = 1.01 ± 0.388 s). In the right triceps brachii muscle in expert basketball players the coefficient of variation (CV) amounted to 44.60% at 81% efficiency, and in novices to 27.12% at 53% efficiency. The time of arm muscle activation during a free throw and its fluctuations vary along with the training experience of basketball players. In all studied groups of players, the variability of muscle activation time in accurate free throws is greater than in inaccurate free throws. Free-throw speed is irrelevant for free-throw efficiency.

## Introduction

The outcome of a basketball match can be decided on the free-throw line, and uniform free-throw shooting is a vital part of the game. Most basketball players perform multiple free-throw shooting routines during training in order to develop a uniform shooting technique^1^. Initially, however, the basketball players need to focus on the understanding of the optimal conditions of free-throw performance so they should know the degree of ball release angle, where the ball should be aimed, how much backspin should be put on the ball, whether it is advantageous to release the ball higher above the ground, how to make the throw quickly, and other key elements necessary for the mastery of the most effective free-throwing technique^2^.

Free throws are always made from the same distance from the basket, and during their execution the shooter does not have to be concerned with differentiating the amount of throwing force and with watching out for defenders’ actions. Basketball coaches usually emphasize that the free-throw movement pattern should be uniform^3^. The idea is to make free-throw preliminary movements as uniform as possible, i.e. placing hands on the ball, bringing the ball to the forehead level, flexing the joints of the upper and lower extremities, and propelling the ball by stretching the joints, and making sure free-throws are performed by the shooters in the same way, all of which can greatly affect shot success. However, if free throws are all performed in the same way, the question remains why not all free throws are accurate? Where can there be a fault in this uniform approach?

Many authors have been studying free throws in basketball in order to improve players' technical skills and overall team performance. There is a general acknowledgment that preshot preparation carried out by most shooters before the free throw execution has a significant positive impact on shot success^4,5^. It was also recognized that improved postural control and lower mean center-of-mass (COM) speed when executing a free-throw resulted in increased shot accuracy^6^. The same studies showed that variability may facilitate or hamper a successful free throw, which requires further investigation. Tran and Silverberg^2^ also studied the optimal conditions of ball release during a free throw. It was found that the shooter should release the ball 7 feet above the ground, place up to 3 Hz of backspin on the ball, aim the ball towards the back of the ring, and launch the ball at 52° to the horizontal. Ideally, free-throw shooters should release the ball as high above the ground as possible as long as it does not adversely affect their launch consistency. Also the shooters’ movements during the free-throw performance should be as smooth as possible. Hamilton and Reinschmidt^7^ examined the optimal free-throw trajectory in basketball and concluded that the initial angle and speed should be 60° and 7.3 ms, respectively. An analysis of a physical free-throw model revealed that the optimal free-throw conditions varied from player to player, not only because of the players’ different body height but also because of their different levels of joint angle-velocity consistency^8^, thus it would be necessary to design a separate model for each player. Using high-speed cameras Ogawa et al.^9^, investigated the relationship between free-throw accuracy and anthropometry, physical fitness tests and performance variables. They showed that basketball players with a shorter and less fluctuating pre-shot routine, i.e. time taken, minimum angle when taking the ball back, angle at ball release, angular displacement during the forward arm swing, and angular velocity at ball release on the elbow, shoulder, hip, knee, and ankle, demonstrated a higher accuracy of free throws. The accuracy of free throws can also be affected by the self-efficacy of free-throw performance^10^. Uchida et al.^11^ in their study of free-throw success prediction by novice and elite basketball players showed that when observing a shooter executing a free throw elite players anticipate a score or a miss much better than the novice players. It should also be noted that the effectiveness of free throws can be influenced by player's fatigue^12^, but not necessarily in high-level players^13^.

A successful basketball player must realize the best ways to coordinate his or her body movements in each game situation. Novice players tend to exhibit rigidity in their movements, while expert players perform their movements more smoothly and in a more unconstrained way. The learning process, on the other hand, leads to the release of degrees of freedom, which is linked with adaptations of the motor system to change^14^. The variability of dynamic coordination patterns can be seen as a necessary indicator of fluctuation, which enables the adaptation of a movement pattern from one situation to another^15^. Individuals perform and acquire movements differently^16–18^. Functional or compensatory variability is assumed to be one of the criteria of specialist knowledge, thanks to which experienced players can increase stability at the macro level and decrease stability at the micro-level. The organization and enhanced stability of individual movement patterns are associated with higher advancement levels^18^. Experienced players compensate for the combined movements to achieve the same goal despite different initial conditions. The direction of the compensation is from proximal to distal, and a higher variability occurs in more distal joints^19^.

The success of a free-throw shooter depends ultimately on two factors. First, the shooter must understand what a desired shot should look like, which does not always have to be optimal. The other, no less significant factor is the shooter's consistency. The actual shot will most likely differ from the free-throw shooting pattern due to the inevitable variation of the latter. The correct choice of the pattern and standard deviations of release conditions completely determine shot success^2^. Optimal release conditions during free throws are not taken for granted since they are interrelated. For example, the ball release height affects the optimal launch angle, which in turn affects the aiming spot. Due to these multiple factors, a large number of free throws need to be studied in different ways in order to gain a full understanding of the optimal release conditions.

When analyzing such a complex research task as free-throw shots, many effects are to be expected as they depend on the shooter’s individuality and skills, and the interpretation of empirical results can be difficult^20^. The research on free throws in basketball indicates a compensatory variability between the joint movements of the shooter's throwing arm. At least six different joints are involved in the execution of a free throw, from the foot joints to the hand joints. By default the onset of the free-throw always involves some disturbances which must be compensated for. Compensation will be primarily expected in the distal joints, therefore they should be the focus of prospective research. During free-throws novice players can exhibit a greater variability in the trajectory of free throws than more experienced shooters, which is probably due to the shooters’ constant seeking the optimal position of the arms before the throw^21^.

Moreover, free-throw efficacy can be also determined by quiet eye training^22^, HR_max_ below 80%^23^, stable arousal and a relatively constant amount of attention to the task prior to movement execution^24^, sleep extension to 10 h^25^, compensatory behavior between the joints of their free-throwing arm on the basis of proprioception^26^, and synergy or a coupling between the elbow and wrist angles^27^.

Basketball is one of the most popular team sports in the world. It is played by millions of people, but so far there have been no studies of muscle bioelectrical activity during a free throw, and it is the muscles that are the last component that propels the basketball player's body and thus the ball. This type of research can significantly contribute to the understanding of what factors can affect free-throw effectiveness. During a free throw, the release power is generated by both lower and upper limb muscles. However, the movement of the ball and its accuracy are primarily determined by the precise work of the shooter's upper limbs muscles, which gives the ball its final speed and backspin. Is it possible to establish for how long these muscles should be activated for a free throw to be effective?

Using surface electromyography (sEMG) the present study aimed to measure the activation time of the arm muscles in basketball players with different skill levels, which contribute to the success of the free throw. In the study the impact of basketball practice on players' skill levels was not examined directly, but rather individual differences in the organization of movement system during a free throw were explored. The results of the study may contribute to a better comprehension of the critical elements of a free throw, improve players' skills as well as coaches' understanding of movement, facilitating the selection of the best players. By setting an optimal movement pattern, the study will indicate the best way to perform successful free throws. The main purpose of this research is to answer the question of how to direct the body's movements properly in order to achieve the best possible effectiveness of the throw, or what the effect of the muscle activation time is on the efficiency of the throw. More specifically, to find out how muscle activation affects differences in basketball players' efficacy, with more shot efficiency expected in advanced basketball players. It was also hypothesized that novice players would experience greater variability of muscle activation, and that this variability will change along with players’ training and competitive experience.

## Results

The overall percentage of accurate free throws in the experiment was 69 ± 17.3%. The expert players made successful shots with the mean efficiency of 81%, i.e. 8% (p = 0.379) more accurate than the intermediate-level players, and 28% (p = 0.014) more accurate than the novices. Intermediate-level basketball players made 20% more accurate shots than novices (p = 0.059).

**Figure 1:**
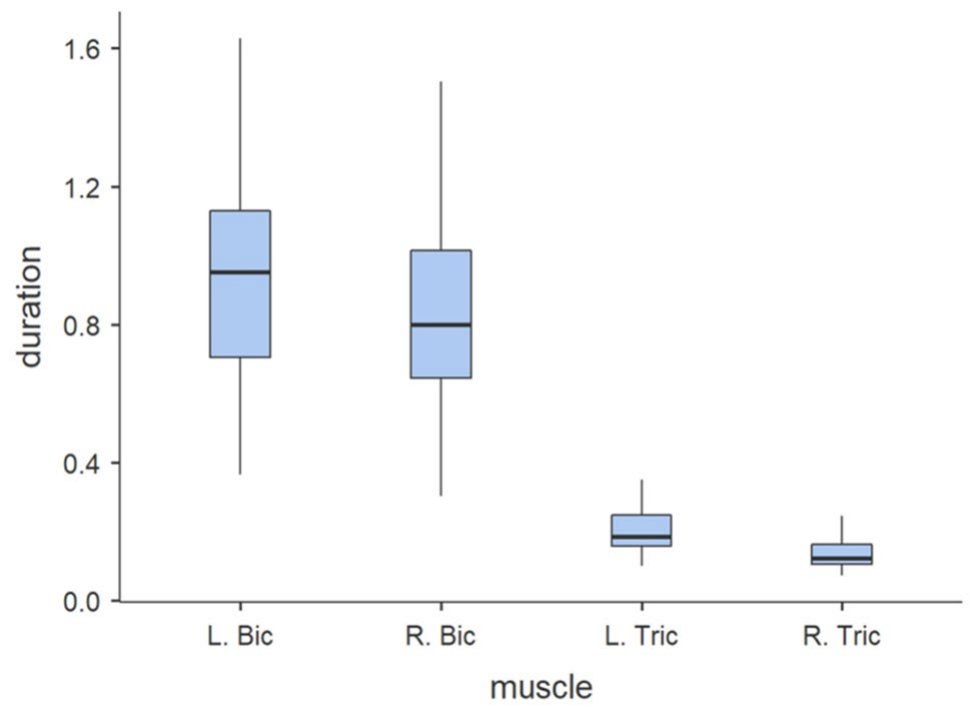
Duration of muscle activation time during the free-throw [s].

The study assessed the free-throw time based on measurements of arm muscle activity. In each of the right-handed basketball players the sequence of muscle activation was identical. The first to activate were the muscles responsible for bringing the ball over the shooter's head. The muscle activation time (t) in the earliest activated muscle, i.e. the left biceps brachii (L. Bic), was t = 0.942 s before the release, followed by the right biceps brachii (R. Bic) t = 0.832 s (Fig. 1). After reaching the appropriate release height, in order to release the ball, the shooters activated the muscles responsible for extending the arms: first the left triceps brachii muscle (L. Tric) t = 0.206 s, and last the right triceps brachii muscle (R. Tric) t = 0.142 s.

Free throws, as measured starting from L. Bic., were performed most quickly by the novices, who executed them 0.486 s faster than the intermediate-level players, and 0.346 s faster than expert players (Table 1). The activation of subsequent muscles followed the same order as L. Bic. Additionally, in both biceps brachii muscles significant inter-group differences in free-throw time were found (p < 0.05).

**Table 1:** Duration of muscle activation time during the free-throw [s] (mean ± standard deviations), in groups.

Variable	Group	L. Bic	p	R. Bic	p	L. Tric	p	R. Tric	p
Mean ± SD	Experts	1.01 ± 0.388	0.038*	0.925 ± 0.359	0.035*	0.206 ± 0.0814	0.561	0.139 ± 0.062	0.151
	Intermediate	1.15 ± 0.146		1.02 ± 0.153		0.235 ± 0.0823		0.174 ± 0.0529	
	Novices	0.664 ± 0.225		0.549 ± 0.233		0.178 ± 0.0769		0.111 ± 0.0301	
Coefficient of variation	Experts	38.42%		38.81%		39.51%		44.60%	
	Intermediate	12.70%		15.00%		35.02%		30.40%	
	Novices	33.89%		42.44%		43.20%		27.12%	

* Statistical significance >0.05.

The highest variability in muscle activation during a free throw performance was noted in expert players, i.e. 40.34% per muscle, followed by 23.28% in intermediate-level players, and in the novices 36.66%. Only in the R. Tric. muscle the highest value was recorded for expert players and the lowest for novices, which corresponds to the increase in the effectiveness of free throws along with players’ training experience.

**Figure 2:**
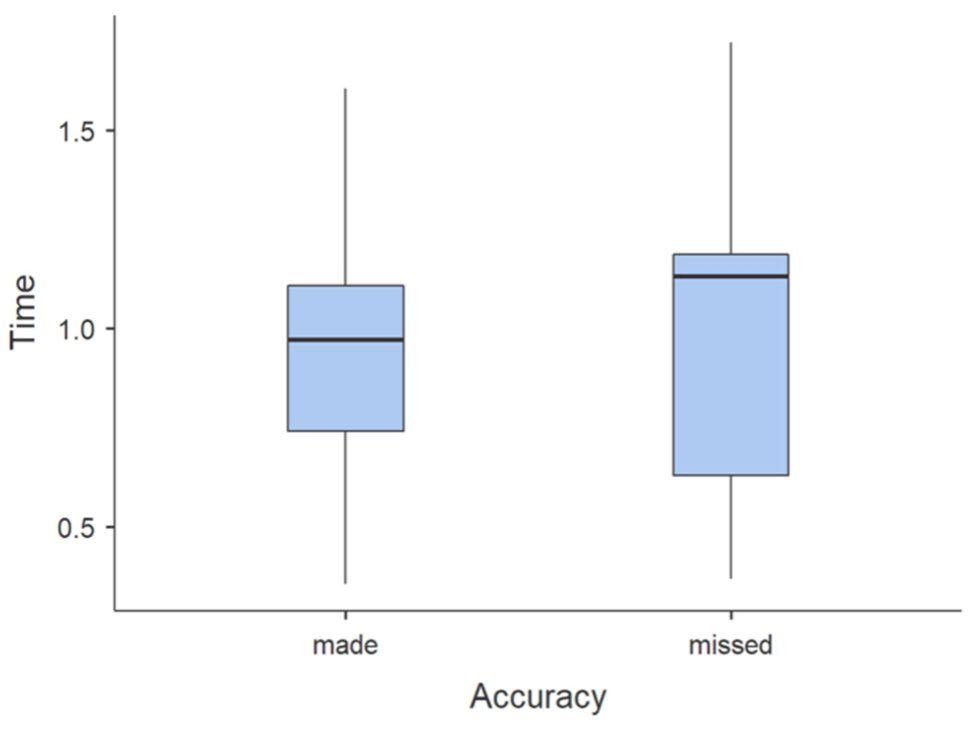
Time of free throw made and missed.

The activation time of the measured muscles during accurate shots was 0.013 s shorter than during inaccurate shots. The muscles also had 5% less variability of muscle activation, with no significant differences between them p > 0.05 (Fig. 2).

Expert basketball players, when making an accurate free throw (Table 2), released the ball 0.18 s faster (L. Bic.) and 0.11 s faster (R. Bic.), while it took them more time to throw the ball after the activation of L. Tric. for 0.037 s, and R. Tric. for 0.28 s when making an inaccurate shot (Table 3). Intermediate-level basketball players, while making successful free throws, released the ball more slowly (by 0.02 s as measured for L. Bic., by 0.01 s for R. Bic. and L. Tric., and by 0.03 s for R. Tric). The novices, on the other hand, made an accurate free throw by releasing the ball more slowly (by 0.114 s for L. Bic., 0.116 s for R. Bic.), but by 0.004 s faster for L. Tric., and by 0.042 s for R. Tric.

**Table 2:** Duration of muscle activation time during the free-throw made [s] (mean ± standard deviations).

Variable	Group	L. Bic	p	R. Bic	p	L. Tric	p	R. Tric	p
Mean ± SD	Experts	1.01 ± 0.373	0.038*	0.93 ± 0.354	0.038*	0.206 ± 0.0814	0.21	0.141 ± 0.0616	0.075
	Intermediate	1.16 ± 0.151		1.03 ± 0.138		0.237 ± 0.0919		0.178 ± 0.0646	
	Novices	0.692 ± 0.225		0.583 ± 0.244		0.161 ± 0.0749		0.0928 ± 0.0365	
Coefficient of variation	Experts	36.93%		38.06%		39.51%		43.69%	
	Intermediate	13.02%		13.40%		38.78%		36.29%	
	Novices	32.51%		41.85%		46.52%		39.33%	

* Statistical significance >0.05.

**Table 3:** Duration of muscle activation time during the free-throw missed [s] (mean ± standard deviations).

Variable	Group	L. Bic	p	R. Bic	p	L. Tric	p	R. Tric	p
Mean ± SD	Experts	1.19 ± 0.38	0.011*	1.04 ± 0.32	0.013*	0.169 ± 0.0339	0.18	0.113 ± 0.0278	0.038*
	Intermediate	1.14 ± 0.158		1.02 ± 0.19		0.236 ± 0.0697		0.175 ± 0.0382	
	Novices	0.578 ± 0.136		0.467 ± 0.124		0.165 ± 0.0469		0.135 ± 0.0469	
Coefficient of variation	Experts	31.93%		30.77%		20.06%		24.60%	
	Intermediate	13.86%		18.63%		29.53%		21.83%	
	Novices	23.53%		26.55%		28.42%		34.74%	

* Statistical significance >0.05.

The variability of activation of all muscles during accurate free throws was 39.55% in experts, 25.37% in intermediate-level players, and 40.05% in novices. In the case of inaccurate throws the variability was lower and amounted to 26.84% in expert players, 20.96% in intermediate-level players, and 28.31% in novices, respectively. In all groups of players the time of accurate free throws did not differ significantly from the time of inaccurate free throws (p > 0.05).

A positive correlation between basketball players’ body height and body mass r = 0.901 (p < 0.001), and a moderate positive correlation between body height and free throw time was found for L. Bic. r = 0.531 (p = 0.042) and R. Bic. r = 0.561 (p = 0.029). Additionally, there was a positive correlation (r = 0.96; p < 0.001) between L. Bic. and R. Bic., and between L. Tric. and R. Tric. (r = 0.911; p < 0.001).

## Discussion

Free throws are an important part of a basketball match. The margin of victory in a basketball match can be very small and a one-point free throw can decide the match outcome. Since free throws are always executed from a particular place on the court, the shooter must release the ball within five seconds, and there is no pressure from the opposing players, they are usually the most effective shots during a basketball match. And since free throws can decide the match outcome the question remains why professional basketball players do not perform them more effectively. Additionally, we know that in order to improve free-throw effectiveness, a player makes a lot of throws in training sessions in order to reach a proper, repeatable throwing pattern, and the effects could often be better. According to the results of a number of studies a great deal is known about optimal free-throw conditions. However, the free-throw remains such a key activity in basketball that it constantly requires further and innovative research, especially that in the case of many players their free-throw effectiveness is far from satisfactory.

The aim of this study was to improve the knowledge of the best possible free throwing technique by determining temporal aspects of arm muscle activation in relation to the skill level of basketball players. The most experienced of the studied right-handed basketball players achieved the best free throw effectiveness, and the novices had the worst results, which confirmed the observed general trend^28,29^. During the preliminary movements before the execution of a free-throw, the right-hand shooters hold the ball with both hands. The right hand is under the ball, as if supporting its weight, while the left hand is placed on the side of the ball. This commonly accepted technique pattern^30^ was no different during the present experiment. Before the free throw, all other joints of the arms and legs were properly flexed and ready to provide propelling force for the shot. There are still questions about how to direct the body's movements properly in order to achieve the best possible effectiveness of the throw, or what the effect of the muscle activation time is on the efficiency of the throw.

There have been no studies of the time and sequence of muscle activation during free throws in basketball. The study of the muscles of both arms shows that right-handed basketball players always shoot a free throw with an identical activation timing: left side muscles are always activated first, followed by the right side muscles. This occurred both in the first phase of the ball movement, i.e. lifting it over the head, where both brachii biceps muscles were activated, and in the phase of moving the ball from above the head forward and propelling the ball towards the basket, where the brachii triceps muscles were activated while straightening the arm. Of course, the antagonistic muscles always cooperated in movement, i.e. if they were not responsible for the movement, they were activated to fulfill the stabilizing function^31^. The muscle activation in the present study refers to the activation of the muscles responsible for movement, occurring immediately before the onset of the dedicated movement^32^. Moreover, it was noted that the activities of right and left side muscles were significantly correlated (r ≥ 0.911), which indicates their nearly synergic effect.

The study revealed significant differences in the duration of the free-throw between the groups of basketball players. The novices shot the fastest free throws, followed by the experts, and the intermediate-level players, who had the longest muscle activation time. The study results also demonstrated significant interpersonal differences in the duration of free throws, which suggests that basketball players use very different techniques to achieve the same goal^8,33^. The free throw effectiveness is determined, the time of muscle activation is crucial for the efficiency of throws, and another important factor related to efficiency is the shooter's ability to adjust the free-throw time.

Considering all basketball players, the time of muscle activation in accurate free throws and their variability was lower than in inaccurate throws, however, the difference was small and statistically non-significant. On the other hand, taking into account the results in each of the three groups of players, it turned out that the time of muscle activation in shooting accurate free throws was more varied than in shooting inaccurate free throws. Additionally, it was the expert basketball players who achieved the best average of accurate free throws and varied the time of all throws the most. Thus the results of a study by Mullineaux and Uhl^27^, in which the observed high variability of free throws was regarded as a compensatory strategy of adapting to throwing technique errors, were confirmed. Therefore, the expert players, through their experience of performing a large number of free throws, model the time of arm muscle activation to achieve greater efficiency. This differs from a study of the center-of-mass (COM) speed demonstrating its decrease along with players' more advanced skills^6^. The results of a study by Ogawa et al.^9^ on a free-throw routine with a shorter and less varied pre-shot time are not applicable to muscle activation time either. Perhaps it is because it focused on a homogeneous group, and only studies on players representing various skill levels can explain the differences in movement patterns^5^.

It is also worth noting that the variability of muscle activation time does not increase with players' experience and skill level, since it was the intermediate-level basketball players who had the lowest coefficient of variation. On the other hand, the high coefficient of variation in the novice players may be caused by the early stages of teaching of motor activities, during which the optimal position of arms before making a free throw is still sought^21^. It is not always the case that high variability of muscle activation time determines high free-throw effectiveness. Thus, the report by Verhoeven and Newell^6^ could be further expanded, since the question whether the variability of movement contributes to or hinders a successful free throw depends on players' advancement level.

The present study also shows that the smallest variation in activation time in expert players is found in proximal muscles and the greatest in distal muscles. In order to achieve high-free throw effectiveness, advanced basketball players compensate for the coordination structures from proximal to distal^19^. This variability of dynamic systems can be seen as a necessary indicator of fluctuation, which allows the motor system to adapt from one situation to another^15^. Since the distal high variability is a determinant of free-throw effectiveness, it is easier to understand why it is the most successful shooters who vary the activation time of the brachii triceps muscle most often in all free throws. Additionally, triceps brachii is the only muscle in which the lower effectiveness of free throws in groups (81%, 73%, 53%) was found, the less varied its activation time was (44.60%, 30.40%, 27.12%). It is worth noting, however, that this is a mean result, not always reflected in individual cases.

Unlike in Ogawa et al.^9^ no significant correlation has been found between free throw efficacy and other variables, body height, body mass and muscle activation time. Thus, the free-throw time was not a direct determinant of free-throw effectiveness, or the shooter's physical conditions. However, a high correlation of body height and body mass with the activation of muscles on the right and left sides of the body was revealed. Additionally, the moderate strength of the correlation concerned the relationship between the activation time of both biceps brachii muscles and body height. The activation time of the biceps brachii muscles affected the time between the onset of their activation and the last contact of the right hand with the ball, so in the case of the present study it had an impact of the free throw time. It follows that the taller the shooter was, the more moderately the activation time increased.

Furthermore, free-throw time patterns were established, which are a key factor in sport for achieving the best results^18^. In the case of free throws in basketball, however, this is not a simple matter as free-throw shooting is a complex activity, and the final outcome is dependent on many factors. In addition, the actual shot will be different from the pattern at the time of its execution^2^ since information continues to flow into the system as the activity takes place, and the movement is then modified.

A limitation of this study is the low number of analyzed throws. It turned out that the number of missed throws was lower than the number of accurate shots. It was particularly visible in the group of expert players with 18 accurate shots out of the total of 20, i.e. 90% effectiveness. Therefore, in future studies it will be necessary to increase the number of attempts to better explain the determinants of free-throw efficiency in basketball. In the study, the impact of basketball practice on players' skill levels was not examined directly, but rather individual differences in the organization of the movement system during a free throw were explored. Also the study only involved members of three male basketball teams. A more reliable prospective research should also focus on groups of female basketball players.

## Conclusions

The free throw effectiveness is determined by the muscle activation time of the free throw and varies between different groups of basketball players. The muscle activation time is the shortest in novice players and the longest in intermediate-level players, while the highest variability of muscle activation was found in elite basketball players. In the groups of studied basketball players, accurate free throws more than inaccurate free throws were associated with a higher variability of muscle activation time. The speed of the free-throw is not significant for free-throw efficiency. The highest variability of the right brachii triceps activation time and free throw effectiveness are found in expert basketball players, and the lowest variability in novice players.

The present research can contribute to a better understanding of the free throwing technique and can make it more effective. It can potentially enhance free-throw training methods that would have a more practical impact on basketball players’ free-throw performance. It is highly likely that players need to shoot as many free throws as possible during basketball training as this allows them to gain experience and vary their muscle activity time better in order to make a successful shot.

In the future, we will expand our research to include the analysis of players' other muscles, whose monitoring could contribute to an even better understanding of the proper activation of muscles in free throws. We also believe that it is worthwhile to scientifically examine players by grouping them in terms of their position on the court, or body height. It is also recommended to broaden the research scope and include other types of throws or field goals in order to set the optimal throwing conditions during a real sports competition.

As a result of this research we recommend, that suggestions of coaches and also free throws of players should not artificially reduce or extend the muscle activation time of a free throw. The activation time of a free throw is an individual matter, and it is more important to adjust the control engine, which manifests itself in the increased variability of muscle activation in the most effective players. Speculating, the increased variability of activation in the most effective basketball players is most likely a result of the number of throws made during training.

## Material and methods

### Participants and study design

The study involved three teams of basketball players of different experience levels: experts, intermediate-level players, and novices. The team of experts played in the 1st Polish basketball league and the youth groups were the leading teams in leagues at the regional level. Five examined players from each team were the starters and represented each basketball position (n = 15). The information about the players’ age, body height and body mass can be found in Table 4.

**Table 4:** Characteristics of examined group (mean ± standard deviations).

Group	Age (years ± SD)	Height (cm ± SD)	Weight (kg ± SD)	Experience (years ± SD)
Experts	28.4 ± 3.6	190.8 ± 9.4	86.2 ± 6.3	10.2 ± 1.3
Intermedite	20.6 ± 1.5	183.6 ± 11.2	73.1 ± 13.9	5.4 ± 0.7
Novices	15.8 ± 0.7	172.2 ± 8.0	55.8 ± 8.2	1.8 ± 0.5

Each player was to perform 20 free throws, made after passing the ball from the partner who was under the basket. All participants were right-handed and shot from the right hand. The time of the throw was measured with digital high-speed cameras synchronized with sEMG. The measurement apparatus consisted of two cameras with the image resolution of 2048 × 1088 pixels and frame rate of 250 fps, a synchroniser, a computer, a Noraxon MT 400 electromyograph, and StreamPix 5 ProAnalyst software.

### Study design

The bioelectric activity of the muscles was recorded with a sampling rate of 1000 Hz. Ag/AgCl electrodes were placed on the pretreated skin surface of the player’s body. The sEMG records of 20 free throws executed by each player were analyzed with MyoResearch XP Clinical Application Protocols. Four pairs of electrodes were placed between muscle–tendon junctions of the right and the left biceps brachii muscles (R. Bic. and L. Bic.) and the right and the left triceps brachii (R. Tric. and L. Tric.) as recommended by SENIAM. Two image-capturing high-speed cameras were placed: one at the front and one at the side of the measuring station, to enable the precise recording of players’ movements.

The duration of the free throw was assessed using detection of EMG signals from the arm flexors and extensors, with the help of visual assessment of the investigator. The onset of the bioelectrical tension of the muscle was determined from 300 ms before the occurrence of movement^32^, when the bioelectrical signal increased by 3 SD. The result was the time from the detection of bioelectrical arousal in the muscle until the moment of ball release, i.e. one frame after the last contact of the shooter’s hand with the ball.

Prior to the experiment, the players were asked to complete a short survey questionnaire, and no player reported taking any caffeine-based supplements or substances containing guarana or creatine. The players were asked to refrain from caffeine intake from 6:00 p.m. prior to the test, and to refrain from vigorous exercise as well as to maintain normal dietary habits within 48 h prior to the test. The trials were performed during the regular evening training session, and were preceded with a warm-up.

The participants had no health contraindications or injuries within the upper limbs. All the participants were informed about the potential risk related to the examination and were informed about the purpose and the course of tests. Informed consent was obtained from all participants and/or their legal guardians. Study was approved by the Bioethical Commission of the Chamber of Physicians in Opole No. 206, in accordance with the guidelines specified in the Declaration of Helsinki on human experimentation.

### Statistical analysis

The collected data were subsequently subjected to statistical analysis using Jamovi 1.2 software (retrieved from https://www.jamovi.org). Due to the lack of normality of the distributions and homogeneity of variance of the analyzed variables, nonparametric analysis tools were applied. To determine the level of the significance of the differences, the non-parametric Wilcoxon test was used to determine the dependencies between the samples. Correlations were calculated with the rho Spearman test. The level of significance of differences was set at p ≤ 0.05.

## Data Availability

The datasets generated and/or analysed during the current study are not publicly available. However, the data are available from the corresponding author on reasonable request.
